# MicroRNA-22 Impairs Anti-Tumor Ability of Dendritic Cells by Targeting p38

**DOI:** 10.1371/journal.pone.0121510

**Published:** 2015-03-31

**Authors:** Xue Liang, Yu Liu, Shiyue Mei, Miaomiao Zhang, Jiaxuan Xin, Yuan Zhang, Rongcun Yang

**Affiliations:** 1 Department of Immunology, Nankai University School of Medicine, Tianjin, China; 2 Tianjin Key Laboratory of Ionic-Molecular Function of Cardiovascular Disease, Department of Cardiology, Tianjin Institute of Cardiology, Second Hospital of Tianjin Medical University, Tianjin, China; 3 State Key Laboratory of Medicinal Chemical Biology, Nankai University, Tianjin, China; 4 Key Laboratory of Bioactive Materials, Ministry of Education, Nankai University, Tianjin, China; Istituto Superiore di Sanità, ITALY

## Abstract

Dendritic cells (DCs) play a critical role in triggering anti-tumor immune responses. Their intracellular p38 signaling is of great importance in controlling DC activity. In this study, we identified microRNA-22 (miR-22) as a microRNA inhibiting p38 protein expression by directly binding to the 3’ untranslated region (3’UTR) of its mRNA. The p38 down-regulation further interfered with the synthesis of DC-derived IL-6 and the differentiation of DC-driven Th17 cells. Moreover, overexpression of miR-22 in DCs impaired their tumor-suppressing ability while miR-22 inhibitor could reverse this phenomenon and improve the curative effect of DC-based immunotherapy. Thus, our results highlight a suppressive role for miR-22 in the process of DC-invoked anti-tumor immunity and that blocking this microRNA provides a new strategy for generating potent DC vaccines for patients with cancer.

## Introduction

Dendritic cells (DCs) are the most effective antigen presenting cells (APCs) with exclusive function of activating naïve T cells through presenting antigens to them [1]. They express high levels of MHCII molecules to exhibit antigens efficiently, and then activate CD8^+^ and CD4^+^ T cells. Besides that, DCs can also interact with natural killer (NK) cells and B cells to forge a bridge between innate and adaptive immune systems [2,3]. Thus, they have been considered as the primary activator of immune response and are closely involved in inflammation, autoimmune disease, transplantation immune response and so on. For a long time, researchers have been focusing on their ability to induce the reactions of T cells and B cells. However, in recent years, the anti-tumor function of DCs has been attracting more and more attention [4].

DCs play an important role in anti-tumor immune responses, while on the other hand, tumor cells can reciprocally secrete some soluble factors, including TGF-β, IL-10, etc, to disrupt the differentiation of DCs and their ability to activate immune responses, to fight back, which may be the crucial barrier holding back tumor treatment [5,6]. These tumor-derived factors interrupt the regular function of DCs by activating several intracellular signals, such as MAPK, JAK/STAT and NF-κB pathways. It has been recently reported that the dysfunction of DCs caused by tumor cells is accompanied by excessive activation of MAPK signaling pathways [7]. Thus, studying MAPK signals can lay a foundation for directly or indirectly mitigating tumor cells’ damage on DCs. As an important member of MAPK family, p38 plays a role in regulating various cell activities and is considered to be the joint center of signal transduction. Regulating the expression and function of p38, therefore, can be an effective method to improve DC-related tumor treatment.

MicroRNAs, as small non-coding RNAs, widely distributed in various species are able to elaborately regulate expression of genes related to various physiological and pathological processes including immunity responses [8]. As to DCs, miRNAs are indispensable in regulation of their development, differentiation and functions. This may be seen via the actions of let-7i, miR-142-3p, miR-146a, the miR-148 family, miR-155, and miR-155* in regulating cytokine production in response to DC activation, and as an inherent characteristic of DCs via constitutive miR-146a expression [9]. Since DCs help to orchestrate immune responses by secreting appropriate cytokines and influencing CD4^+^ T cell subset differentiation [9], miRNAs may offer the foundation for modifying them to improve immune responses against tumors.

MicroRNA-22 (miR-22), originally isolated from HeLa cell line, has been found to be ubiquitously expressed in various tissues [10–12]. Evolutionary clustering suggests that miR-22 is highly conserved in vertebrate evolution, indicating its functional importance in vertebrate species. It has been deduced from the statistical analysis of 3’ untranslated regions (3’UTRs) in transcriptome that miR-22 participates in the regulation of many target genes [13].

Here, we have found that miR-22 could be expressed in dendritic cells and proved that miR-22 can impair the tumor-suppressing function of DCs and directly bind to the 3’UTR of p38 mRNA to down-regulate p38 protein. The decreased expression level further interferes with the synthesis of DC-derived IL-6 and the differentiation of DC-driven Th17 cells.

## Materials and Methods

### Mice, cell lines and murine bone marrow derived dendritic cells

Four- to six-wk-old female C57BL/6 mice (Beijing Animal Center) were maintained in a specific pathogen-free animal facility for at least 1 wk prior to use. The animal experiments were performed in accordance with institutional guidelines and the study was approved by the ethics committee of Nankai University.

The following cell lines were purchased from American Type Culture Collection: murine monocyte/macrophage RAW264.7, murine melanoma B16 and human embryonic kidney 293T. 293T cells were cultured in Dulbecco’s modified Eagle’s medium (DMEM) supplemented with 10% fetal bovine serum (Hyclone) at 37°C in a humidified 5% CO_2_ atmosphere. RAW264.7 and B16 cells were maintained in the same condition except that the basic culture medium was RMPI 1640 rather than DMEM.

Murine bone marrow derived dendritic cells (BMDCs) were generated from C57BL/6 mice. Mice were sacrificed and their bone marrow cells were collected by removing the femurs and tibia, cutting off each end, and flushing out the bone marrow with PBS using a syringe. The pooled cells were harvested by centrifugation at 600 ×g for 10 min and the erythrocytes contained were removed by incubation with Ammonium Chloride lysing buffer for 5 min at room temperature. The cells were then washed in RMPI 1640 medium supplemented with 10% fetal bovine serum. BMDCs were obtained by culturing these bone marrow cells for 5–6 days in 500 U/mL GM-CSF (R&D Systems). The percentage of CD11c^+^ dendritic cells (DCs) in the final cell population was 70–80% by fluorescence activated cell sorting (FACS) analysis.

### Surface and intracellular staining

Single cells were prepared from the removed mouse inguinal lymph nodes and 10^6^ cells/well were cultured in 24-well plates. They were re-stimulated for 6 h with 50 ng/mL PMA, 1 μg/mL Ionomycin and 0.66 μL/mL GolgiStop Protein Transport Inhibitor (BD Pharimingen). Then surface and intracellular staining was performed using the mouse Th1/Th2/Th17 phenotyping Kit (BD Pharmingen) according to the manufacture’s instructions. Stained cells were analyzed with CellQuest software on an FACSCalibur (BD Biosciences).

### Prediction of microRNA-binding sites

The predicted microRNA binding sites were downloaded from TargetScan 5.1 Mouse (http://www.targetscan.org/mmu_61/) [14].

### Real-time RT-PCR

Total RNA was isolated using TRIzol Reagent (Invitrogen). 1 μg of RNA was reverse transcribed with the M-MLV Reverse Transcription System (Invitrogen) according to the manufacture’s instructions. PCR was performed using the SYBR Green Real-time RT-PCR Master Mix plus (TOYOBO) as described by the manufacture. PCR primers used here were as follows. GAPDH: 5′-TGCACCACCAACTGCTTAG-3′ (sense), 5′-GATGCAGGGATGATGTTC-3′ (antisense); p38: 5′-ACAAACCAAGTCATCAAGG-3′ (sense), 5′-ATCAGAAGGAACCACACT-3′ (antisense); IL6: 5′-AACGATGATGCACTTGCAGA-3′ (sense), 5′-GAGCATTGGAAATTGGGGTA-3′ (antisense). Amplification was performed by denaturation at 95°C for 10 min, followed by 40 cycles of 95°C for 30 sec, 58°C for 30 sec and 72°C for 30 sec. GAPDH was performed on each experimental sample as an endogenous control. The real-time RT-PCR was carried out in a Bio-rad IQ 5 Multicolor real-time RT-PCR system and their software was used to calculate the cycle threshold of each reaction. All reactions were run in triplicate.

### Transfection

RNA mimics, inhibitor and negative control oligonucleotide were purchased from RiboBio (Guangzhou, China). Cells were transfected with the indicated oligonucleotides (100 nM) using the Entranster-R system (Engreen Biosystem) according to the manufacturer’s instructions.

### Western blotting (WB)

Cells were lysed in lysis buffer (Beyotime) containing complete protease mixture (Sigma-Aldrich). After centrifugation, the lysates were boiled in SDS loading buffer and resolved by 12% acrylamide gel electrophoresis in the presence of SDS (SDS-PAGE), then transferred to a PVDF membrane (Millipore) and probed with 1:1000 dilution of a rabbit anti-p38 or anti-beta-actin polyclonal antibody (Santa Cruz Biotechnology), followed by 1:1000 dilution of a horseradish peroxidase-conjugated goat anti-rabbit secondary antibody (Promega). Then the polypeptides were revealed using ECL reagent (Amersham Biosciences). All of the figures illustrating Western blotting analyses are representative of at least three independent experiments.

### Enzyme linked immunosorbent assay (ELISA)

After stimulation with LPS at a concentration of 1 μg/mL for 24 h, cell supernatants of different groups were collected and analyzed using the Quantikine ELISA Kit for IL-6 (R&D Systems, Minneapolis, MN) according to the manufacturer’s instructions.

### Luciferase reporter assay

To get the recombinant luciferase mRNAs, the DNA of the 3’ untranslated region (3’UTR) of mouse p38 mRNA or its mutant, changing the 8nt binding site for microRNA-22 (miR-22), was amplified by PCR using modified primers (sense: 5'-AACTCGAGCGAGTCCTCTCCTAGGACTA-3', and antisense: 5'-TTGCGGCCGCACACAAAGCTTAAATATG-3' for both the wild-type and mutant 3’UTRs. As for the mutant one, 2 additional primers were required to generate the mutant site from the original wild-type template. One was paired with the aforementioned sense primer: 5'-TCAGTGTCATCGACGGGGGGGGTGGGG-3', and the other was used together with the antisense one: 5'-CCACCCCCCCCGTCGATGACACTGAATC-3'. Then the 2 kinds of products were mixed to perform an overlapping PCR experiment and the final product was the expected mutant 3’UTR.), digested with *Xho* I and *Not* I. Then each of them was cloned into the vector siCHECK-2, downstream of a luciferase CDS, respectively. The validity of these constructs was verified by sequencing.

293T cells were seeded in 24-well plates and cotransfected with 1 of the 2 constructs combined with dsRNA control or dsRNA mimics of miR-22 using Lipofectamine 2000 Reagent (Invitrogen). After 24 h of incubation, the cells were collected for application in the Luciferase Reporter System (Promega, Madison, WI) following the manufacturer’s instructions. All the luciferase reporter assays were repeated 3 times within each experiment.

### Tumor challenge model

B16 melanoma cells were maintained as mentioned above and injected s.c. into C57BL/6 mice to form solid tumors. 10^6^ of these cells were administrated to each mouse. Then the mice were monitored for tumor growth every 2 days.

When solid tumors formed and their volumes could be measured by calipers 12 days after inoculation, mice were randomly divided into several groups to receive intratumoral DC injection. Differentially treated DCs were administrated to different groups respectively at a number of 10^6^ per mouse every 7 days. In the meantime, analgesic steps were also performed to minimize the suffering of the mice by a subcutaneous Buprenorphine (Temgesic, 0.05 mg/kg) treatment every 12 h, and hence their condition was also monitored at this frequency from then on.

Humane endpoints were introduced into the study and mice were euthanized by cervical dislocation method when their smallest tumor volume reached to 3000 mm^3^. Tumor size was measured in two dimensions by calipers and determined by the following formula: Width^2^×Length×π/6, where Width was the lesser value of the two dimensions. However, as to the survival study, the criterion to implement euthanasia to a mouse was that it exhibited a moribund state, and the period between tumor inoculation and this endpoint was collected as its survival time. A mouse would be conceived as being moribund if it exhibited the characteristics of severe mobility loss, hunched back, piloerection, ruffled fur and weight loss, and then it would be sacrificed humanely.

## Results

### Identification of the microRNAs targeting p38 gene

**Fig 1:**
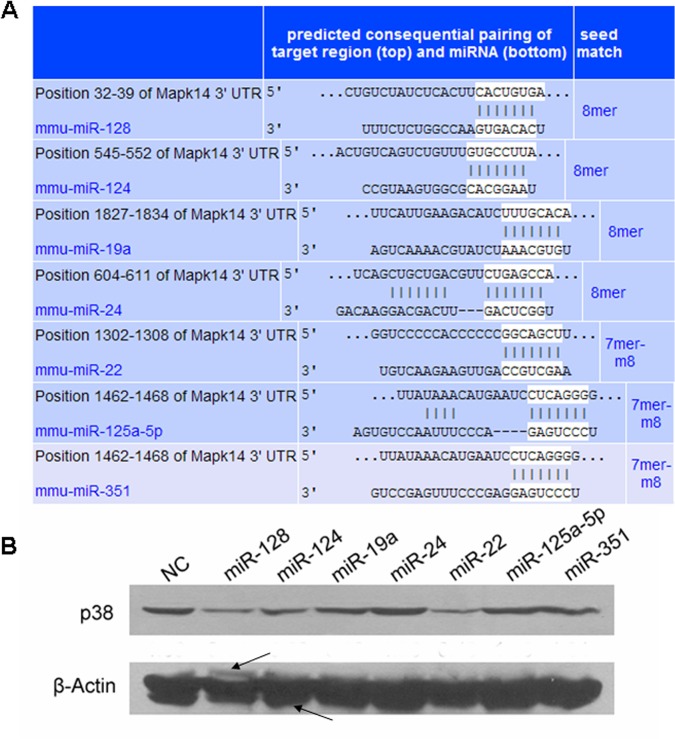
Identification of the microRNAs targeting p38 gene. (A) The potential microRNA binding sites in the 3’ untranslated region (3’ UTR) of p38 gene were predicted using TargetScan Mouse and the result showed 7 microRNA candidates, including microRNA-128 (miR-128), miR-124, miR-19a, miR-24, miR-22, miR-125a-5p and miR-351. (B) microRNA mimics corresponding to the aforementioned candidates were transfected to RAW264.7 cells respectively and the p38 protein levels after 24 h of transfection were detected by Western blotting (WB) to validate the prediction result experimentally. 3 out of the 7 microRNAs, including miR-128, miR-124 and miR-22 showed significant negative effect on p38 protein expression. This figure is representative of 3 independent experiments. It should be illustrated that, in this WB result, although the p38 bands are single and clear, the β-actin bands are not so clear and some noise signals, like the ones marked by the arrows, exist around them. This may be due to the non-specificity of the anti-β-actin antibody used. However, the main β-actin bands are still obvious and have much intense signal than the non-specific ones.

Dendritic cells (DCs) from patients with cancer are functionally defective, while p38 MAPK signaling pathway play an important role in regulating function of dendritic cells in tumor environments [7]. In order to identify the microRNAs regulating the expression of p38 gene and therefore with the potential to control DC activity, we searched the possible microRNA binding sites in the 3’ untranslated region (3’UTR) of p38 mRNA by TargetScan Mouse (http://www.targetscan.org/mmu_61/) [14]. The result showed that there were 7 potential microRNAs targeting p38 gene, including microRNA-128 (miR-128), miR-124, miR-19a, miR-24, miR-22, miR-125a-5p and miR-351 (Fig 1A). We next investigated whether these miRNAs could silence their potential targeting molecule p38. We transfected the selected microRNA mimics into monocyte/macrophage cell line RAW264.7 cell line, which is often used as a cell model to study intracellular signaling patwhay in dendritic cells [15–17], and then measured the expression level of p38 protein by Western blotting (WB). The result suggested significant decreases in the protein expression after the transfections of miR-22, miR-124, and miR-128 (Fig 1B).

### miR-22 inhibits the translation of p38 gene by binding to the 3’UTR of its mRNA

**Fig 2:**
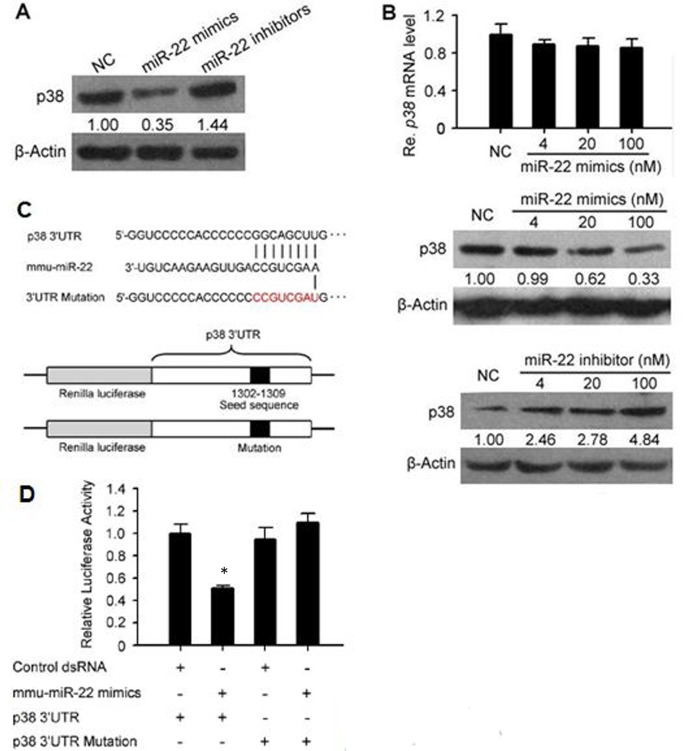
miR-22 suppresses the expression of p38 gene on the post-transcriptional level by binding to the 3’ UTR of its mRNA and inhibiting translation. (A) miR-22 mimics and inhibitor showed reverse effects on p38 protein expression. When p38 protein in RAW264.7 cell line was detected after 48 h of transfection of miR-22 mimics, inhibitor or negative control oligonucleotide, miR-22 mimics displayed a negative effect while inhibitor showed a positive effect on the expression of p38 protein compared with the control oligonucleotide. (B) Although miR-22 mimics had a significant influence on p38 protein level, real-time RT-PCR after 24 h of transfection of RAW264.7 didn’t reveal any effectiveness of this microRNA on p38 transcription. Hence, the significant negative effect of miR-22 on p38 protein level as revealed by the anti-correlation between the band gray scale and the dose of the mimics administered, was derived from inhibiting mRNA translation rather than promoting its degradation. On the other hand, as the dose of miR-22 inhibitor loaded on RAW264.7 cell line increased, p38 protein level showed an up trend, further illustrating the suppressive effect of miR-22 on p38 expression. (C) To find the miR-22 binding site on p38 mRNA, two kinds of recombinant luciferase mRNA were constructed by replacing the original luciferase mRNA 3’UTR with p38 mRNA 3’UTR, wild-type or mutant. The exact 8nt binding sequence for miR-22 on p38 mRNA was kept or mutated to facilitate or disrupt the binding of miR-22. (D) The 8nt sequence was proved to be necessary to mediate the inhibitory effect of miR-22. The reporter plasmid with wild-type or mutant 3’UTR of p38 was transfected together with miR-22 mimics or control oligonucleotide. After 24 h, wild-type group showed a significant decrease in the luciferase activity, indicating that miR-22 mimics had functioned to inhibit the translation of the recombinant mRNA. However, if the 8nt sequence was mutated, as in the mutant group, the inhibitory effect disappeared. This rescue phenomenon suggested that p38 mRNA was a direct target of miR-22 dependent on the 8nt sequence. All the WB results shown in this figure are representative of 3 independent experiments. The real-time RT-PCR and luciferase reporter experiments were also repeated at least 3 times. Their bar plots are expressed in the form of mean±SD. * means a significant decrease appears when compared with the control group (p-value < 0.05, t test).

Since all of the 3 valid microRNAs showed negative effects on the expression of p38, we inferred that they were likely to act as dendritic cell regulators to affect anti-tumor responses. Previous data had shown that miR-22 could be highly expressed in mouse DCs and induced in DC progenitor cell cultures with GM-CSF [18]. Especially we had found that miR-22 could be up-regulated by tumor [19]. Thus, we further investigated the effect of miR-22 on the dendritic cells. We had reported that this microRNA was also contained in the monocyte/macrophage cell line RAW264.7 [19], so we wanted to further clarify the effect of miR-22 to p38 gene using RAW264.7. We firstly transfected the miR-22 mimics and inhibitor (chemically synthesized oligoenucleotide, which could specifically bind with miR-22 and inhibit its function) into RAW264.7 cell line respectively, followed by measuring the p38 protein expression level by WB. The result indicated a significant expression decrease after the transfection of miR-22 mimics as compared with the control group, and a reverse result after the transfection of inhibitor (Fig 2A). Under the transfection of miR-22 mimics of different doses, the transcriptional level of p38 mRNA were found to be similar measured by real-time RT-PCR, while the expression level of p38 protein, measured by Western blotting, decreased following the increase of dose. In contrast, as the dose of inhibitor rose, the expression level of p38 protein increased (Fig 2B). These results suggested that miR-22 regulated the expression of p38 gene post-transcriptionally by inhibiting mRNA translation rather than promoting its degradation.

Since miR-22 functioned on the post-transcriptional level to inhibit p38 mRNA translation, in view that some microRNAs exerted this effect by binding to the 3’UTRs of the target mRNAs, we constructed a recombinant mRNA to test whether p38 mRNA was a direct target of miR-22 by replacing the 3’UTR of luciferase reporter gene with the 3’UTR of p38 gene, wild-type or mutant, in the plasmid siCHECK-2. Mutant 3’UTR included an 8nt change in the original miR-22 binding site (Fig 2C). These plasmids were transfected into 293T cells respectively in pair with miR-22 mimics and the fluorescence intensities were analyzed after 24 h. The recombinant luciferase gene with wild-type p38 3’UTR was significantly inhibited by miR-22 mimics, while the one with mutation still kept the normal activity (Fig 2D). This result indicated that miR-22 inhibited the translation of p38 gene by binding to its 3’UTR directly.

### miR-22 is expressed and functions in dendritic cells

**Fig 3:**
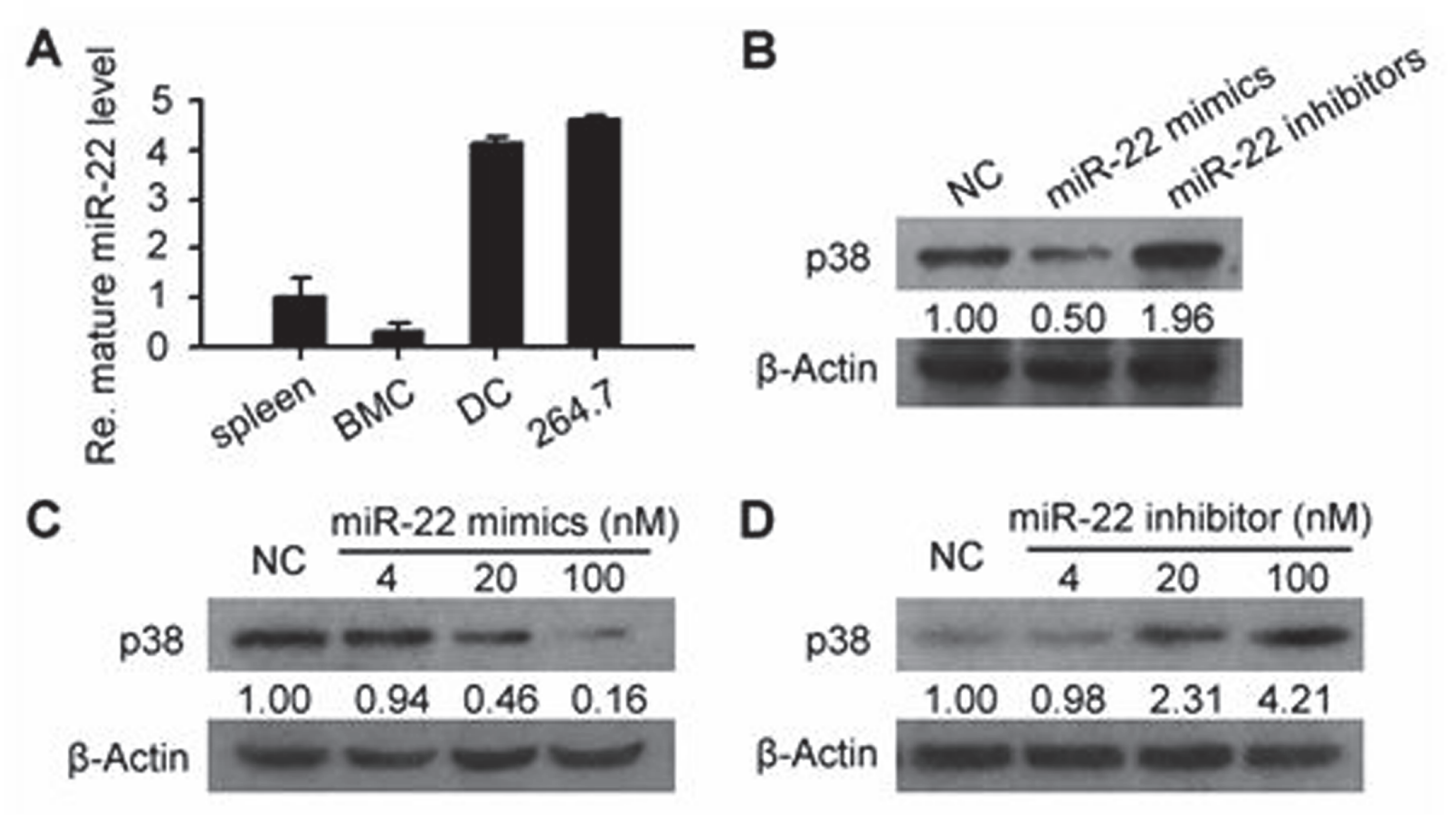
miR-22 is expressed and functions in dendritic cells (DCs). (A) miR-22 expression level was examined using real-time RT-PCR and this microRNA was found to be distributed in a wide range of cells, including mouse splenocytes, bone marrow cells (BMCs), BMC derived DCs and murine monocyte/macrophage cell line RAW264.7. The great increase of miR-22 level after BMCs being induced to DCs was reminiscent of a close relationship between this mircoRNA and the process of DC differentiation. (B, C, D) Similar to the RAW264.7 model, miR-22 exhibited an inhibitory effect on p38 protein expression in DCs. After 48 h of miR-22 mimics or inhibitor transfection, p38 levels were probed by WB. The gray scale of p38 band was negatively correlated with the dose of miR-22 mimics while positively correlated with the dose of miR-22 inhibitor. All the experiments in this figure were performed at least 3 times. The real-time RT-PCR result in (A) is expressed as mean±SD.

Consistent with our previous report [19], the expression of miR-22 was not limited to RAW264.7 cell line, as shown by our real-time RT-PCR result. Mouse splenocytes, bone marrow cells (BMCs) and BMDCs also had an internal level of it. Notably, although the miR-22 content was low in murine BMCs, once they were induced *in vitro* into BMDCs, the amount of miR-22 would increase significantly. It was not clear whether it was the cause or the result of DC differentiation, but it was affirmative that miR-22 had a close relationship with the process of DC development (Fig 3A). Thus, to better illustrate the general function of miR-22 in dendritic cells, we next transfected the mimics or inhibitor of miR-22 into DCs and detected the protein level of p38. Similar to the case of RAW264.7 model, miR-22 exhibited an inhibitory effect on this target gene, the expression level of p38 decreased in the presence of the mimics while increased in the presence of the inhibitor (Fig 3B). The correlation or anti-correlation between p38 protein level and the dose of miR-22 inhibitor or mimics as revealed by the dose response further confirmed this effect (Fig 3C and 3D).

### **miR-22 regulates the expression of IL**-6 in **DCs**

**Fig 4:**
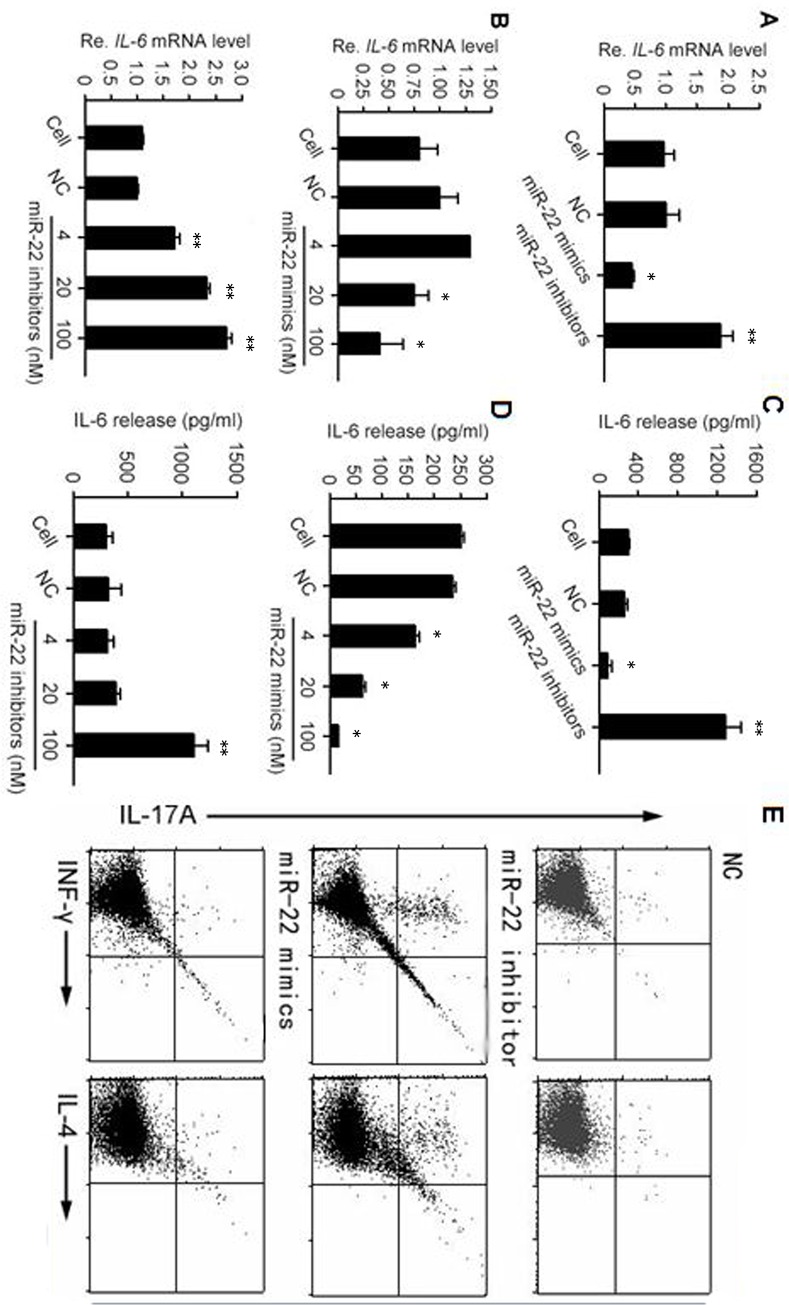
miR-22 inhibits DCs from expressing IL-6 and driving Th17 cells differentiation. (A) Based on the facts that p38 had a stimulatory influence on the transcription of IL-6 gene in bone marrow derived DCs (BMDCs) and that miR-22 could prohibit p38 mRNA from being translated, it was inferred that miR-22 mimics and inhibitor would hinder and promote the expression of IL-6 respectively. This was confirmed by the real-time RT-PCR result of the BMDC groups loaded with different reagents after 24 h of LPS stimulation. The result also illustrated the effect of miR-22 appeared as early as the transcription event occurred. (B) Dose effects of miR-22 mimics and inhibitor were examined and the results further confirmed the negative effect of mimics and positive effect of inhibitor on IL-6 mRNA transcription. (C) Inhibitory effect of miR-22 on IL-6 expression could also be detected on the protein level. BMDCs were grouped and treated as mentioned above and their culture supernatants were collected to determine the IL-6 protein concentrations using enzyme linked immunosorbent assay (ELISA). (D) Dose series of miR-22 mimics and inhibitor were integrated into the BMDC transfection procedure and IL-6 protein levels were examined by ELISA. The results further confirmed the inhibitory effect of miR-22 on IL-6 protein expression. (E) miR-22 inhibitor promoted the ability of DCs to induce Th17 cells generation *in vivo*. C57BL/6 mice bearing B16 melanomas were given DC immunotherapy using differentially transfected BMDCs, and then the T cell populations in the tumor microenvironment were detected by flow cytometry. All the cells shown in this figure were gated from CD4^+^ lymph cells and it was obvious that compared with the control and miR-22 mimics groups, mice injected intraltumorally with miR-22 inhibitor loaded DCs exhibited a higher percentage of CD4^+^ IL-17A^+^ Th17 in their inguinal lymph nodes. All the real-time RT-PCR and ELISA experiments included in this figure were performed at least 3 times. The bar plots are expressed in the form of mean±SD. * means a significant decrease appears when compared with the control group (p-value < 0.05, t test), while ** represents a significant increase relative to the control (p-value < 0.05, t test).

As one of the most important members of the MAPK family, p38 had been reported to exert impacts on the process of tumor generation through its effects on the activities of some inflammation associated interleukins, including the pivotal IL-6 factor [20]. As to the context of dendritic cell intracellular environment, it had been reported that p38 had a positive effect on IL-6 expression in DCs [21]. This effect of p38 on IL-6, as well as the aforementioned relationship between miR-22 and p38, promoted us to further explore whether mR-22 could regulate the production of IL-6. Mouse BMDCs were transfected with miR-22 mimics or inhibitor followed by being treated with LPS and then were collected to examine the IL-6 mRNA level with real-time RT-PCR. The result showed that, compared with the control group, the transcripts of IL-6 decreased significantly under the influence of miR-22 mimics, and increased remarkably after inhibitor transfection (Fig 4A). Additionally, the dose effects of miR-22 mimics and inhibitor were also explored and the mRNA change tendency validated the former result (Fig 4B). It could be inferred from the negative effect of miR-22 on p38 protein level and the stimulatory influence of p38 on IL-6 expression that miR-22 mimics and inhibitor would reduce and enhance the IL-6 mRNA concentration respectively. This was consistent with our experimental results and also been supported by a publication concerning the negative influence of miR-22 on IL-6 in rat and cell models of cerebral ischemia-reperfusion injury [22].

To further advance our conclusion to the IL-6 protein level, we conducted an IL-6 enzyme linked immunosorbent assay (ELISA). A much lower concentration of IL-6 protein was detected in the DC supernatant of the mimics group compared with the control group. While as to the inhibitor group, the concentration increased significantly (Fig 4C). Also, dose response experiments were performed and the change tendencies were similar to the real-time RT-PCR results except that the ELISA plot of the inhibitor dose series had an inflection point prominently lagged behind the one on its corresponding mRNA plot (Fig 4D). This hysteresis might be attributed to the different processes to produce a functional protein and a mature mRNA. A secretory protein like IL-6 needed a complex and time-consuming process to achieve its maturation and correct localization, including post-translational modification, transport between different cellular organs, and so on, while the case of its mRNA was relatively compact [23].

### miR-22 inhibitor can stimulate Th17 cell generation through IL-6

The relationship between miR-22 and IL-6 reminded us that miR-22 might function in many physiological and pathological processes through the mediator IL-6. As to the immune system, IL-6 contributes to host defense against pathogens, but dysregulation of IL-6 production plays a significant pathological role in various autoimmune and inflammatory diseases [24]. The most prominent function of IL-6 was to regulate the balance between IL-17 producing Th17 cells and regulatory T cells (Treg) [25]. Together with TGF-β, IL-6 could induce the development of Th17 cells, who was a key player in the pathogenesis of autoimmune diseases and protection against bacterial infections, from naïve T cells, while inhibit TGF-β induced Treg differentiation [26]. In view of this function, we inferred that miR-22 could also inhibit Th17 cell differentiation and this was verified by the following experiments. We established the B16 mice tumor model and then performed a DC immunotherapy by injecting the BMDCs transfected with miR-22 mimics, inhibitor or negative control oligonucleotide intratumorally to the mice. When the experimental period ended, the inguinal lymph nodes nearest to the mice tumors were removed to detect Th1/Th2/Th17 cell populations by fluorescence activated cell sorting (FACS). As expected, the proportion of Th17 cell population of the inhibitor group was much greater than the other two groups, suggesting a stronger ability of miR-22 inhibitor loaded DCs to induce Th17 cell differentiation *in vivo* (Fig 4E).

### miR-22 impairs the tumor-suppressing ability of DCs

**Fig 5:**
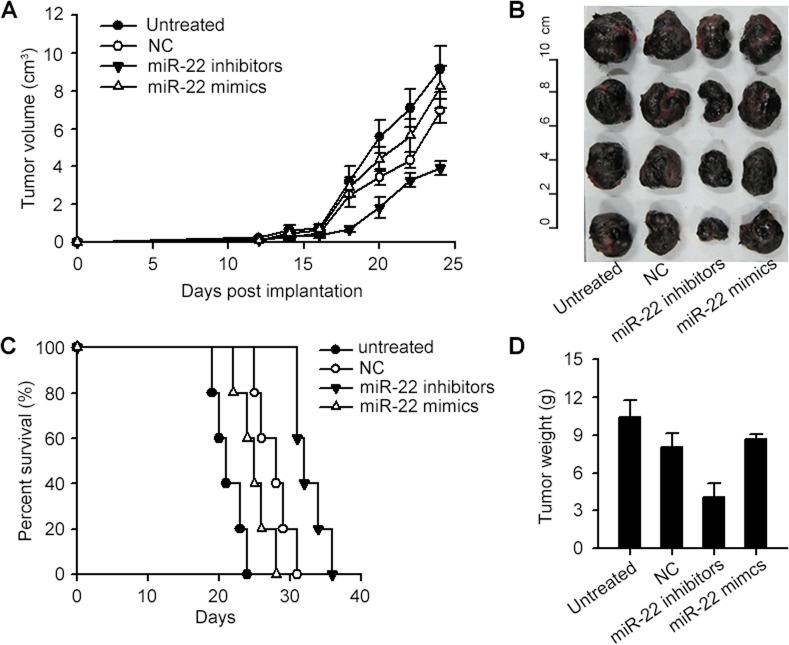
miR-22 inhibitor enhances the anti-tumor ability of DCs. The curative effects of the immunotherapy using differentially treated DCs were evaluated in 4 aspects including tumor growth plot (A), final tumor volume (B), animal survival time (C) and final tumor weight (D). The miR-22 mimics treated DC group showed the weakest therapeutic results. Except for the untreated group, it had the fastest tumor growth rate, the largest tumor volume, the shortest animal survival time and the heaviest tumor weight. On the other hand, the miR-22 inhibitor transfected DCs had the strongest interference on tumor development. The original effect of negative control DCs was enhanced when miR-22 inhibitor was added. These results suggested the tumor-suppressing function of miR-22 inhibitor. All the groups involved in this figure contained 5 mice receiving the indicated treatment. The mice in untreated group were injected intratumorally with PBS instead of DCs, while the other 3 groups were injected with DCs transfected with miR-22 mimics, miR-22 inhibtor and negative control oligonucleotide respectively. The plots of (A) and (D) are expressed as mean±SD.

The original purpose of our study was to find microRNAs with the ability to suppress the p38 gene and develop a feasible tumor therapy strategy by virtue of these RNAs. Hence, the curative effect of miR-22 related oligonucleotides bearing DCs on the B16 mice tumor model was our central focus. The experimental results showed that miR-22 exhibited a tumor promoting effect and impaired the effectiveness of immunotherapy. The mice of miR-22 mimics group had the fastest rate of tumor development (Fig 5A), the largest size of tumor (Fig 5B), the shortest survival time (Fig 5C) and the heaviest tumor weight (Fig 5D) in all the DC treated groups. In contrast, the curative effect of the miR-22 inhibitor group was the strongest of all (Fig 5). The original effect of negative control DCs was enhanced when miR-22 inhibitor was added. These results prompted us to make a conclusion that in DCs, miR-22 could suppress p38 gene expression and further impair the ability of DCs to interfere with the tumor growth, while miR-22 inhibitor could invert this effect and act as a potential immunotherapy reagent.

## Discussion

Dendritic cells (DCs) are the most important antigen-presenting cells (APCs) that bridge innate and adaptive immunity by triggering the activation and differentiation of naïve T cells. They express a repertoire of pattern recognition receptors (PRRs) that sense microbial pathogen products and endogenous ligands to initiate a signaling cascade that culminates in the activation of DCs and adaptive immunity [27]. Among the central pathways regulating this process, the MAPK pathways involving ERK, JNK and p38 play a critical role [28], and human interventions in them may play a part in enhancing the curative effect of DC vaccines for patients with cancer. In this study, we have focused on the p38 MAPK because of its importance in facilitating DC functions, especially in inducing the secreation of cytokine IL-6 and differentiation of Th17 cells.

To intervene in this molecular target in DCs, we adopted a microRNA strategy and firstly screened the endogenous microRNAs related to p38. Based on the TargetScan Mouse prediction and actual experiments, we identified microRNA-22 (miR-22) as a negative regulator of p38 protein expression through directly binding to the 3’UTR of the mRNA. And as we expect, this endogenous microRNA exerts a negative influence on DC functions via silencing p38, which is consistent with the reports demonstrating the critical role of p38 in DC maturation and that p38 inhibition leads to the down-regulation of CD40, CD80 and CD86 markers, raising the risk of DC defunctionalization [29].

Overexpression of miR-22 mimics had an inhibitory effect on IL-6 mRNA and protein synthesis in DCs while miR-22 inhibitor had a reverse influence. This impact on IL-6 level further changed the Th17 cell proportion in tumor microenvironment. It is a significant phenomenon to DCs because initiating Th17 response is one of the most important functions of them. This process is initiated by the C-type lectin-like receptors (CLRs) coupling to signaling via the kinase Syk and the adaptor CARD9 [30]. The significant influences of miR-22 mimics and inhibitor on this DC function hinted the importance of this microRNA in DCs. Consistent with our result was the report that murine splenic DCs with p38 deletion showed a selectively lower IL-6 expression than those of wild-type mice after immunization *in vivo* or LPS stimulation *in vitro*. This impairment of IL-6 level further led to the weakened ability of these DCs to drive the expression of IL-17A and IL-23R in T cells because IL-6 was the most potent positive regulator of Th17 polarization [21]. Follow this axis, the inhibitory effect of miR-22 to p38 in DCs made miR-22 mimics a negative regulator and miR-22 inhibitor a positive regulator of both IL-6 expression and DC-induced Th17 differentiation as revealed in our study.

This effect on IL-6 and Th17 partially contributed to another function of DCs influenced by miR-22, their anti-tumor ability, which is our primary research focus. It is well-known that a controversy has been existed for a long time around the question of whether IL-6 and IL-17A are anti-tumor or tumor promoting factors. This contradiction between the 2 standpoints is likely to have a close relationship with the various experimental conditions and a specific conclusion may only be suitable for its specific condition. As to the B16 tumor challenge model, which is adopted in this study, existing reports have concluded that both IL-6 and IL-17A function as anti-tumor factors and can enhance the immune response [31,32]. It proves the rationality of our results demonstrating the higher tumor clearance efficiency of miR-22 inhibited DCs with up-regulated IL-6 and Th17.

MiR-22 was an endogenous tumor-promoting factor. It could be seen from the fact that miR-22 mimics transfected DCs exhibited a weaker therapy effect than the normal ones. The original tumor-suppressing ability of DCs was impaired under the influence of miR-22. This hinted that inhibition of endogenous miR-22 could be a strategy to release more active p38 and DC anti-tumor activity and miR-22 inhibitor could be served as a promising reagent to improve the performance of the existing DC-based therapeutic tumor vaccine.

In summary, this study demonstrates that miR-22, as an endogenous microRNA of DCs, could suppress the translation of p38 mRNA and further down-regulate the expression of IL-6, which in turn interferes with Th17 cell development in tumor microenvironment. This could explain in part the negative effect of miR-22 mimics and positive influence of miR-22 inhibitor on the anti-tumor ability of DCs. These phenomena also suggest that miR-22 could be treated as a novel target of intervention and blocking miR-22 could be a promising strategy to improve the performance of DCs in immunotherapy. Of course, further preclinical studies are needed to test the applicability of this approach to prepare DC vaccines for patients with cancer.

## Supporting Information

S1 Materials and Methods Supporting Information on ethics statement, mice and tumor challenge model. Detailed description on ethics statement, mice and tumor chanllenge model involved in this study.
(DOC)
