# Supplementary material for: MicroRNA-22 Impairs Anti-Tumor Ability of Dendritic Cells by Targeting p38
Source: PLoS One. 2015 Mar 31;10(3):e0121510. doi: 10.1371/journal.pone.0121510 (PMC4380340; doi:10.1371/journal.pone.0121510)
Supplement: S1 Materials and Methods — Detailed description on ethics statement, mice and tumor chanllenge model involved in this study. (DOC) [file pone.0121510.s001.doc]

**Supporting Information**

**Materials and Methods S1.**

**Ethics statement**

The animal experiments were performed in accordance with institutional guidelines and the study was approved by the ethics committee of Nankai University.

**Mice**

Four- to six-wk-old female C57BL/6 mice (Beijing Animal Center) were individually housed in wire- and filter-topped Macrolon typeⅡcages with sawdust bedding under a 12-h dark/light cycle (lights on at 6:30 AM) at a temperature of 22℃ ± 3℃ in specific pathogen-free environment. Cages were cleaned once weekly.

Food pellets and acidified tap water (HCl, pH 2.5-3) were provided to them. At the late stage of tumor challenge experiment, mice could not reach their food and water easily due to tumor growth. Then, they were provided with wet food pellets in the cage. These were refreshed daily to avoid bacterial growth.

**Tumor challenge model**

B16 tumor cells were maintained as mentioned above and injected s.c. into C57BL/6 mice to form solid tumors. 10^6^ of these cells were administrated to each mouse. Then the mice were monitored for tumor growth every 2 days.

When solid tumors formed and their volumes could be measured by calipers 12 days after inoculation, mice were randomly divided into 8 groups. Among them, 4 groups (4 mice in each group) were used to measure the tumor volumes and weights, and the other 4 groups (5 mice in each group) received the survival experiment. 4 kinds of therapeutic methods were adopted in both of these 2 experiments, including treatment with PBS, DCs loaded with miR-22 mimics, miR-22 inhibitor and control oligonucleotides. Each group in each experiment received 1 of these methods at a frequency of 1 treatment every 7 days. DCs were administrated at a number of 10^6^ per mouse. In the meantime, analgesic steps were also performed to minimize the suffering of the mice by a subcutaneous Buprenorphine (Temgesic, 0.05 mg/kg) treatment every 12 h, and hence their condition was also monitored at this frequency from then on.

Humane endpoints were introduced into the study and mice were euthanized by cervical dislocation method when their smallest tumor volume reached to 3000 mm^3^. Tumor size was measured in two dimensions by calipers and determined by the following formula: Width^2^×Length×π/6, where Width was the lesser value of the two dimensions. However, as to the survival study, the criterion to implement euthanasia to a mouse was that it exhibited a moribund state, and the period between tumor inoculation and this endpoint was collected as its survival time. A mouse would be conceived as being moribund if it exhibited the characteristics of severe mobility loss, hunched back, piloerection, ruffled fur and weight loss, and then it would be sacrificed humanely.

For the tumor measuring experiment, tumor volumes were measured every 2 days and the data were used to plot the tumor growth curve, while tumor weights were measured only after the mice were sacrificed and the tumors were dissected out. Both the volume and the weight data in each group were expressed as mean±SD.
